# Supplementary material for: Pooling strategies in V1 can account for the functional and structural diversity across species
Source: PLoS Comput Biol. 2022 Jul 21;18(7):e1010270. doi: 10.1371/journal.pcbi.1010270 (PMC9345491; doi:10.1371/journal.pcbi.1010270)
Supplement: S1 Text — (PDF) [file pcbi.1010270.s001.pdf]

**S1 Text** **Analysis on $\chi$.** We define $\chi_\theta$ and $\chi_\phi$ as the measures obtained from the responses to rotating (angle, $\theta$) or drifting (phase, $\phi$) gratings respectively (green and black lines in S3 Fig). We use non parametric tests as both $\chi_\theta$ and $\chi_\phi$ do not follow a Gaussian distribution. We found that $\chi_\theta$ was significantly higher (less complex) than $\chi_\phi$ (one-tailed Wilcoxon signed-rank test) with $p < 0.001$ in all tested conditions. The only exception being for $p_s =$MaxPool $2D_F$ (not shown), as in this case, no network shows cells with $\frac{\mathrm{F}_1}{\mathrm{F}_0} < 1$ in the second layer. It is worth noticing that the median $\chi_\phi$ tends to be much lower when the pooling function includes the feature space, for $p_s =$MaxPool $2D_F$ and $p_s =$MaxPool $2D_S + 1D_F$, than for a simple spatial pooling, $p_s =$MaxPool $2D_S + 2D_F$ (see S3 Fig). To assess the significance of $\chi_\phi$ and $\chi_\theta$ generating more complex-like or simple-like behavior we used a one-tailed binomial test (sign test). We set the null hypothesis $median(\chi_\phi) < -1$ and $median(\chi_\theta) \geq -1$. Indeed, for $p_s =$MaxPool $2D_S$, the median of $\chi_\phi$ is found to be significantly lower than $-1$ in only a couple of conditions, whereas in general it tends to be greater than $-1$. In analogy with the results shown above, for $p_s =$MaxPool $2D_S + 1D_F$ the network significantly generates a majority of complex-like cells $\chi_\phi < -1$. In the case of $p_s =$MaxPool $2D_S + 2D_F$, we found again a dependence of the amount of complex-like cells and $M_s$ with $\chi_\phi$ being significantly less than $-1$ for $M_s \leq 100$ and highly significantly ($p < 0.001$) for $M_s \leq 81$. On the other hand, $\chi_\theta$ is found to be significantly greater than $-1$ for all networks with $p_s =$MaxPool $2D_S + 2D_F$, $p_s =$MaxPool $2D_S + 1D_F$ and $M_s > 49$. This indicates that, for $p_s =$MaxPool $2D_S + 2D_F$and $p_s =$MaxPool $2D_S + 1D_F$ low $M_s$ tends to create highly nonlinear networks with broad tuning to both phase and orientation, while increasing further $M_s$ generates networks with more simple-like behaviors (see Discussion). For $p_s =$MaxPool $2D_S$ and $p_s =$MaxPool $2D_F$, $\chi_\theta$ is significantly greater than $-1$ in all tested conditions. Finally, we found $\chi_\phi$ to be significantly smaller for $p_s =$MaxPool $2D_S + 1D_F$ than $p_s =$MaxPool $2D_S$ in all tested conditions (single-tailed Mann-Whitney U test, $p < 0.05$). Whereas $\chi_\phi$ was significantly smaller for $p_s =$MaxPool $2D_S + 2D_F$ than $p_s =$MaxPool $2D_S$ for all $M_s \leq 100$ and highly significantly ($p < 0.001$) for all $M_s \leq 64$.
