# Supplementary figures and images for: Pooling strategies in V1 can account for the functional and structural diversity across species
Source: PLoS Comput Biol. 2022 Jul 21;18(7):e1010270. doi: 10.1371/journal.pcbi.1010270 (PMC9345491; doi:10.1371/journal.pcbi.1010270)

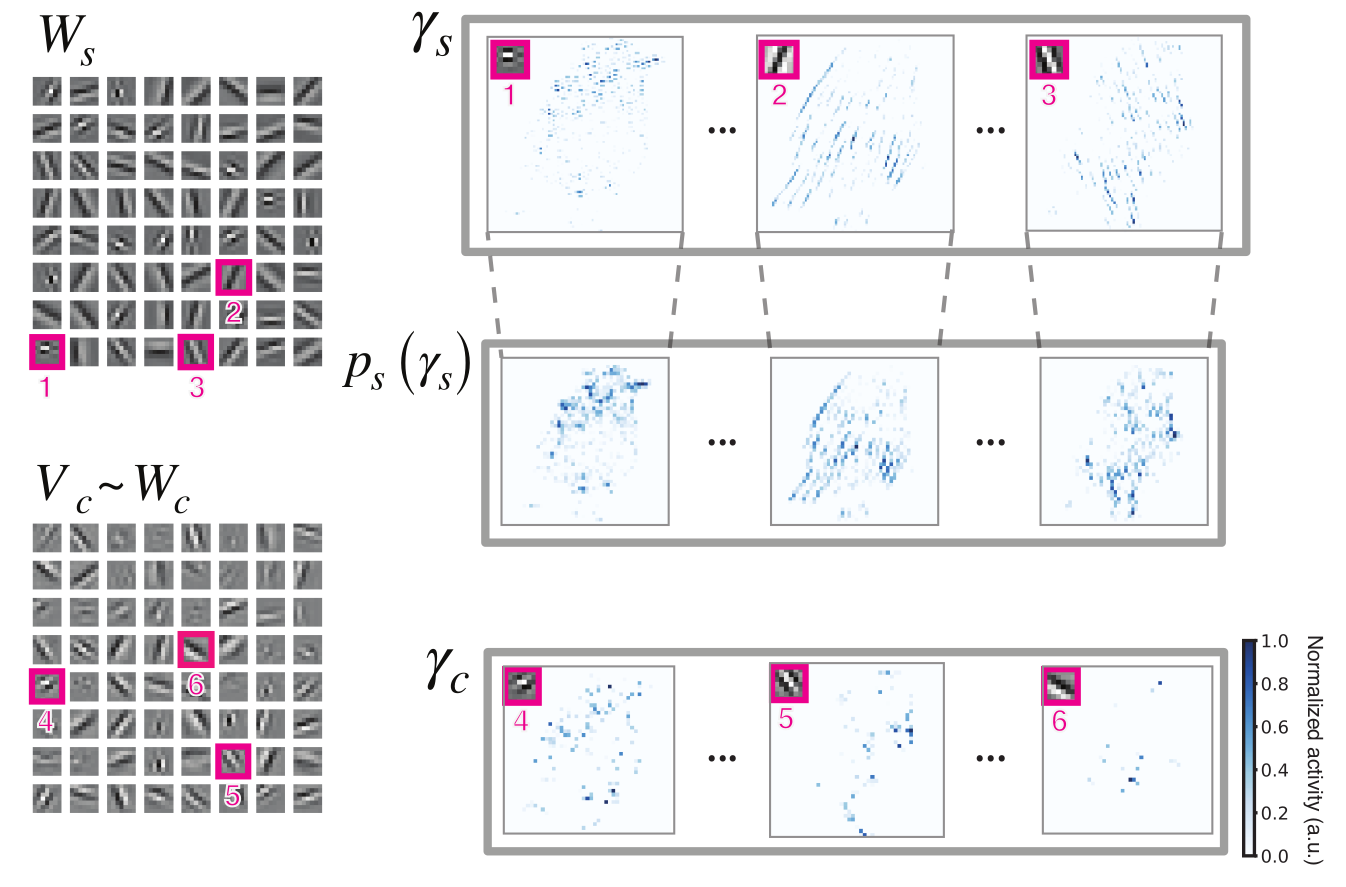

Supplement: S1 Fig — Left. Example of synaptic weights learned from natural images Ws and Wc, for the first and second layer, respectively. Here we show Vc, a linear approximation of Wc (see section ‘Drifting grating vs. Rotating grating’). Each kernel corresponds to a channel in the neural activity maps. Right. Representation of 3 channels from the neural activity maps (γs and γc) elicited by the input x. Here, each pixel represents a model neuron and the color code indicates the amplitude of the neural response (lighter for no response and darker blue for the maximal response, here normalized to 1). The kernel in the top-left corner indicates the preferred stimulus of each channel. (TIFF) [file pcbi.1010270.s002.tiff]

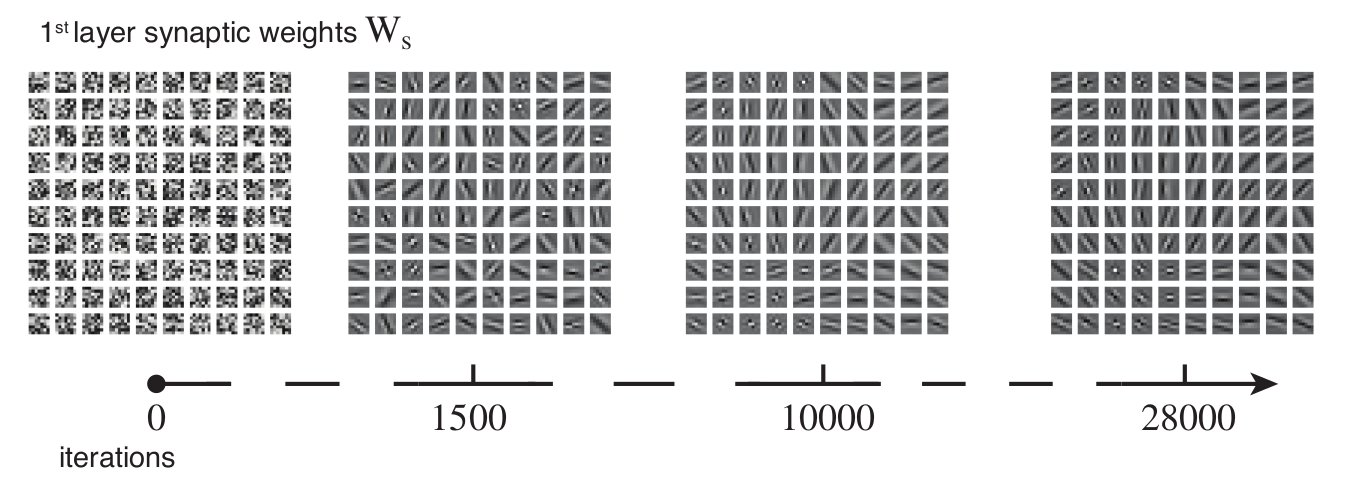

Supplement: S2 Fig — We show the evolution of the topographic organization learned by Ws during training when the SDPC network embeds the MaxPool 2DS + 2DF function. Here, for Ms = 100. The weights are initialized to random values and the network gradually learns from input data. At first, we observe the emergence of edge detectors similar to the ones observed in the V1 of mammals. Gradually, thanks to the combined action of the feedback coming from the second layer of the network and the pooling function in the forward stream, neighboring cells in the topography become tuned to stimuli of similar orientations but different phases, generating a topographically organized map. (TIFF) [file pcbi.1010270.s003.tiff]

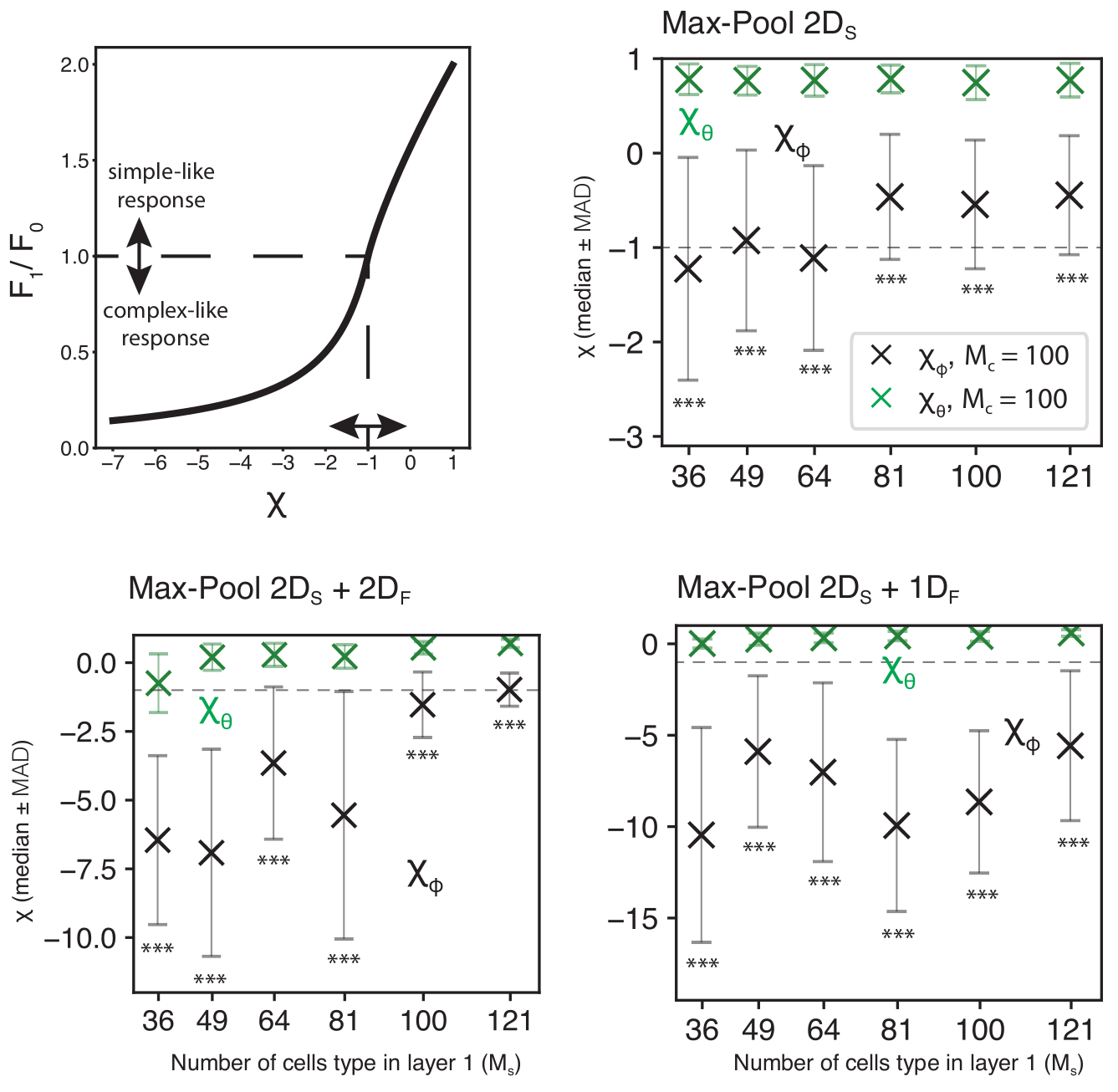

Supplement: S3 Fig — We analyze the same results of Fig 5 in terms of the unimodal variable χ as defined in [39] (see section ‘Modulation ratio and complex behavior’). In the top-left graph we illustrate the nonlinear relationship between χ and F1F0. Here the results for Mc = 100 are shown. To avoid the assumption of normally distributed variables, we represent χ using the median ± MAD (median absolute deviation). Black (χϕ) and green (χθ) lines indicate distributions obtained using drifting and rotating grating, respectively. The dashed lines indicate the value χ = −1 for which F1F0 = 1, the threshold value for which a V1 cell is typically considered either simple or complex. Using drifting gratings as stimuli significantly generated lower values of χϕ (more complex-like cells) than rotating gratings, in all tested settings (one-tailed Wilcoxon signed-rank test). This result confirms that the network’s invariance is linked to the stimulus’ phase and that model cells remain narrowly tuned to orientation. For ps = MaxPool 2DS and ps = MaxPool 2DS + 1DF the distribution of χθ and χϕ do not vary significantly as a function of the network size. When the network shows a functional topographic map, for ps = MaxPool 2DS + 2DF, we can see a clear dependency between χϕ and the number of features in the first layer of the network Ms. (TIFF) [file pcbi.1010270.s004.tiff]
