# Supplementary material for: Pooling strategies in V1 can account for the functional and structural diversity across species
Source: PLoS Comput Biol. 2022 Jul 21;18(7):e1010270. doi: 10.1371/journal.pcbi.1010270 (PMC9345491; doi:10.1371/journal.pcbi.1010270)
Supplement: Attachment — Submitted filename: response_to_reviewers.pdf [file pcbi.1010270.s005.pdf]

Victor Boutin
Inst. Neur. Timone, Aix-Marseille Univ, Marseille, France
victor_boutin@brown_edu

Object: Response to reviewers

Dear reviewer,

We wish to deeply thank both reviewers, as their remarks have allowed us to greatly improve the clarity of the manuscript. The enclosed revised manuscript has been thoroughly amended to answer the comments made by the reviewers. Note that all lines, figures, and equations referenced in this response letter correspond to that in the new version of the article. Please find below a point-by-point description of the changes we have made in the manuscript:

Response to reviewer #1

- **1. Do complex cells also form orientation maps?** Reviewer #1 asked if complex cells also form an orientation map similar to the simple cell map. The formation of orientation maps is mainly due to the combined action of pooling and prediction-error minimization in the first layer. Interestingly, we have observed that orientation maps appear only when there is a feedback signal coming from the upper layer (i.e. the orange-colored connections depicted in Fig 1). As the network has only 2 layers, there is no feedback action on the complex cells' layer, consequently, our network could not account for the emergence of an orientation map in the complex cells' layer. Note that it would be possible to extend the SDPC with more than 2 layers to study if complex cells form orientation maps in this situation. We have added a section in the discussion (section 'Predictive Coding as a mechanism for orientation map formation', from line 311 to 319) to further explain the link between feedback connection and orientation map. We thank the reviewer #1 for pointing out this lack of explanation in our article as it allows us to greatly improve this important point.
- **2. What exactly is shown in the left column of Fig 3, and how does it relate to Fig** 1. In the left column of the Fig 3, we have shown the synaptic weights (i.e. an approximation of neurons' Receptive Fields) of the neurons of the first layer. This is *only* for the first layer, and *all* the synaptic weights are shown (not a subset). To make the link with Fig 1, those RFs are shown on the top-left corners of each of the feature maps in Fig 1b. Concerning the location of neurons, the reviewer should keep in mind that our model is *convolutional*, meaning that the receptive fields we are showing are shared across all positions. Consequently, at a given spatial position, and for a given feature, the neural activity quantifies how strongly the feature is correlated with the input image at the considered spatial coordinate. We have updated the legend of Fig 3 to emphasize the fact that only the features from the first layer are shown.
- **3. Is there a guarantee of convergence of the SDPC?** Indeed, the convexity of the loss function of the SDPC offers a convergence guarantee, but it does not guarantee the SDPC to converge towards a non-trivial solution. In our first experiment, we monitor the shape of the first and second layer Receptive Fields to make sure the network has converged toward a meaningful solution (i.e. a solution with oriented band-pass filters). We have updated the section: 'Pooling in a predictive coding network' (see lines 162-163)

to emphasize this specific point. We thank reviewer #1 for bringing up this potential misunderstanding to our attention.

- **4. Please explain better p_i^{-1} in Eq. 3.** Indeed, p_i^{-1} is the notation we use to denote the approximation of the derivative of the max pooling function (i.e. $\frac{\partial p_i(\gamma_i)}{\gamma_i}$). Note that max-pooling is in general not derivable as there is no defined gradient for non-maximal values. However, the gradient of the maximum with respect to the input is locally linear with slope 1 (only where this input value is maximal). Thus, one can easily approximate the derivative of the max pooling function using an unpooling operator (i.e. a matrix filled with ones for the indices of the maximum elements and with zeros everywhere else). As this unpooling function is the inverse of the max pooling, we have decided to denote it p_i^{-1}. Reviewer #1 correctly raised the fact that we did not explain enough this notation. We have therefore updated the section 'Brief description of the SDPC' (lines 126-128) to make this point clearer.

- **5. In Fig 1, what does the cross in the circle mean? Why is the down-pointing arrow targeting the neuron layer?** The cross with a circle is a symbol borrowed from electronics that illustrates here the computation of the error. The output signal of this error node (i.e. the outgoing arrow) is equal to the sum (or difference if there is a minus in the node) of the input signal (i.e. the incoming arrow). We have updated the legend of Fig 1 to make this point more explicit. The black down-pointing arrow targeting the neuron layer is reflecting the computation described in Eq. 3. The updated neural activity (i.e. γ_i^{k+1}) at layer i is equal to the previous state of the neural activity (i.e. γ_i^k, not shown on Fig. 1) plus a weighted error from the lower level (i.e. $\eta W_i \epsilon_i$, represented by the arrow arriving to the top error node from the left side) minus a weighted error from the higher level (i.e. $\eta \epsilon_{i+1} p_i^{-1}(\gamma_i^k)$, represented by the arrow arriving to the top error node from the right side). To our opinion, the down-pointing arrow is correctly located.

- **6. Is it possible to make the Hebbian rule more obvious in Eq. 4?** We have developed Eq. 4 (which was formerly the Eq. 9) to make the Hebbian update more explicit. The update term is now :

$$-\frac{\partial L}{\partial W_i} = \gamma_i^T \left(p_{i-1}(\gamma_{i-1}) - W_i^T \gamma_i\right) = \gamma_i^T \epsilon_i$$

  While investigating this concern from reviewer #1, we have found a small typo in Eq. 4: the weight update term is not proportional to $\gamma_i \epsilon_i^T$ but rather to $\gamma_i^T \epsilon_i$. This new Eq. 4 makes it clearer that the synaptic weight change is a monotonically increasing function of pre-synaptic and post-synaptic activity, which could therefore be interpreted as a Hebbian update rule. Note that the relationship between predictive coding and Hebbian learning has been thoroughly discussed in [55]. We have added a detailed explanation of the link between predictive coding and Hebbian in the new subsection: 'About the bio-plausibility of the SDPC' (see lines 352-357). We thank reviewer #1 for pointing our attention to this point that is increasing the impact of our message by tightening the link with biology.

- **7. About the sentence: " It only depends on the type of pooling function used ... ".** We agree with reviewer #1 that the formulation of this sentence was indeed unfortunate as it also depends on the input data. We have simply removed this sentence.

- **8. Is it possible to give more details about the map formation mechanism?** Here the map formation could be related to the combined action of two main mechanisms: the pooling (in the feature space) and the predictive coding. The predictive coding scheme

involves minimizing the prediction error between 2 consecutive layers. We observe that when there is no feedback (i.e. no upper layer) then there is no orientation map (not shown in the paper). Consequently, the map emerges as the optimal neural organization to best predict the upper layer's neural activity when a pooling function is introduced in between. As this explanation is important and was indeed missing in the previous version of the article we have added a subsection entitled 'Predictive Coding as a mechanism for orientation map formation' (see lines 311-319). We thank reviewer #1 for pointing out these important details to our attention.

Response to reviewer #2

- **1. About the global narrative of the paper.** We have thoroughly restructured and rewritten the article to make it more focused on our scientific question, which is "*How are orientation maps and complex cells related?*" Given the focus on this question, here are the main modifications we have made on the paper: :
  - We have shortened and reorganized the introduction. In particular, we have drastically reduced the paragraphs on sparse coding and predictive coding as those mechanisms are just properties of the model we use but are not directly related to our original results. We have also reformulated the rest of the introduction toward the link between pooling mechanisms and orientation maps as this is the main point of the article.
  - To facilitate the reading flow, we have included a brief description of the SDPC in the Results section, and we have moved the detailed Method section to the end of the article (as recommended by the journal's guidelines).
  - We have thoroughly reorganized the 'Discussion' section. We have focused the discussion on 4 main parts: 1- the biological interpretation of our results, 2- the link between predictive coding and the map formation mechanism, 3- the relation to other models, and 4- the bio-plausibility of the SDPC.

  We think that this new version is now a perfect fit for the *Plos Computational Biology* journal. To the best of our knowledge, this is the first article showing a model describing both salt-and-pepper and orientation map organization. We deeply thank the reviewer #2 for raising the lack of clarity of the paper narrative in the initial version as we believe the modification has greatly strengthened the impact of this novel revision of the article.

- **2. About differences between our model and the Topographic ICA model [25].** Our work is indeed similar to the Topographic ICA model in the sense that both models account for orientation maps in V1. However, the SDPC differs from the Topographic ICA in 2 ways: in terms of method (the algorithms are not the same) and in the terms of results (the conclusion we draw from the algorithms are different):
  - Method: Topographic ICA models the V1 topology with a distance that quantifies the residual dependency between the components of an ICA. In contrast, our network does not explicitly model the relationship between the features of V1: in the SDPC the topography emerges from the combined action of the optimization process and the pooling function. In lines 325-327, we have updated the article to emphasize this point. We have also added a section entitled 'Predictive coding as a mechanism for orientation map formation' to better explain the link between PC and map formation.

- While topographic ICA only accounts for one combination of cell's type and topology (i.e. the orientation map configuration), our model including SDPC and pooling describes a wider range of configurations (from salt-and-pepper to orientation map). This diversity of emerging configurations is directly originating from the variety of pooling functions that we are using (and leveraged thanks to our convolutional structure). This link between topology and type of pooling function has not been made by Hyvaarinen et *al* in their Topographic ICA article (we have developed this point at lines 327-334).

- **3. Could you better justify the use of convolution in your model?** We agree that the brain is not performing a perfectly organized convolution. Specifically, the weight-sharing mechanism is unlikely to be implemented in the visual cortex. Nevertheless, recent work has shown that convolutional-like representation could be achieved with local untied fully connected networks that avoid replicating the set of weights at each spatial position (see for instance [54]). Importantly, the convolution operation has the advantage to separate the retinotopic space from the feature space. This property is at the core of our analysis as we have used pooling functions that are operating in these two respective spaces. The brain might use a different solution to disentangle the spatial from the feature dimensions (among other dimensions), but convolution still offers a convenient tool to easily differentiate those dimensions. We have updated the section 'About the bio-plausibility of the SDPC' to better justify our use of the convolutional operator (see lines 345-351).

- **4. The pooling operators should be defined earlier.** We agree with the reviewer #2 that the previous organization of the article was making the understanding of the pooling functions in the model description very confusing. Some of the modifications listed in bullet point 1 mitigate this confusion: the pooling function description is now located right after the model description (lines 132-158).

- **5. Could you better justify the use of the max-pooling function?** We agree with reviewer #2 that the argument based on the popularity of the max-pooling in deep learning is not a valid one. Thus, we have removed this argument. The second concern of reviewer #2 is related to the fact that the competition mechanism of max-pooling might just have a duplicate effect with the competition mechanism introduced by sparse coding. Indeed, both sparse coding and max-pooling share a common computational mechanism based on competition. But the crucial difference is that the competition in max-pooling is local (we select the best neurons among a small neighborhood) whereas the competition in sparse coding is global (a minimal number of neurons are selected to predict the input, regardless of their neighborhood). We believe this specific point (local versus global) is making the SDPC even more attractive as it models both simple and complex cells using a similar computational mechanism that has a solid grounding in neurophysiology. We have updated the section 'Pooling functions' (see lines 139-144) to reflect this specific point.

- **6. Wouldn't you want to learn the pooling function?** This would be indeed a major improvement to bring to the SDPC framework. We have briefly discussed the point in the section 'Concluding remarks' (see lines 362-365). The first idea coming to mind is to use the generic activation function described by the following equation (see [38] for more details):

$$y = \frac{\sum_{i=1}^{N} w_i * x_i^p}{k + \sum_{i=1}^{N} x_i^q}$$

This equation describes the neural output (y) as a function of the input (x), their synaptic weight (w_i) and the learnable coefficients p, q and k. Learning these coefficients with back-propagation would allow the activation function to span from energy pooling to max-pooling. We thank reviewer #2 for suggesting such an interesting perspective.

- **7. About the definition of g_i in Eq. 3**. We have now reformulated the loss function (see Eq. 2), such that it is now self-contained and does not rely anymore on g_i.
- **8. Shouldn't the filters in Fig 1b be different?** Reviewer #1 also observed that the representations of the filters in the top left corner of the activity map shown in Fig. 1b were similar. Actually, the filters were not exactly similar yet we agree that the differences were subtle. We have updated the Fig 1b to make sure the filters now appear different from each other. We thank the reviewer #1 for raising this point that improves the overall clarity of our figure.
- **9. Please explain better p_i^{-1} in Eq. 3**. This specific point has also been raised by reviewer #1. Please refer to bullet point #4 in the responses to reviewer #1 for more details.
- **10. What do the W_c features look like?** We have shown some W_c features (see Fig S1). We have decided to keep this visualization in the Supplementary Material section as it is not bringing additional information to our original results. Indeed the W_c are in general really similar to the corresponding W_s features despite the fact that there is a max-pooling in between that is making the second layer highly non-linear. Nevertheless, we have highlighted this a priori surprising point in the section (see lines 170-173).
- **11. About other interesting related work.** We updated the section 'Relation to the state-of-the-art' to include the papers proposed by the reviewer #2 (see lines 335-340). We would like to thank reviewer #2 for bringing these references to our attention.

We hope that, together with the manuscript changes, these answers will sufficiently address the different concerns raised by both reviewers.

On behalf of the authors,

Victor Boutin
